# A Comparison between Gas and Electricity

**Published:** 1920-04-17

**Authors:** 


### HEALTH AND ARTIFICIAL LIGHTING.

### A Comparison between Gas and Electricity.

At the outset of this note it must be made clear that our comments refer particularly to the lighting of living rooms of those small dimensions to which an increasing number of town-dwellers of all classes are perforce becoming accustomed. So often has one heard the paramount hygienic value of the electric system upheld, that at a time when some, at least, of our housing and hospital reconstructions are well on the way and the finer details being put in hand, it is worth while pointing out that there is something of a fallacy in the extreme preference given to electric light when installed in a small apartment.

### A Recent Study.

In framing a final decision on this point, particularly useful information was provided in an address delivered by Samuel Rideal, D.Sc. (Lond.), F.I.C., at the Central Hall, Westminster, in the latter part of last year. At the time, his words undoubtedly corrected many an essential but partially misunderstood conception of principles in hygienic lighting, but from accounts of methods still to be seen in actual progress of preparation one must assume that many responsible organisers have not yet appreciated Dr. Rideal's full meaning, and we may with advantage summarise some of the points which he made. There are great recent changes which must be remembered by the younger workers in health matters, who have perhaps studied from facts and figures relating to conditions of ten or so years ago. Not only have incandescent electric-glow lamps been greatly changed by the introduction of metallic filaments in place of carbon, and gas-filling instead of vacua, but also corresponding improvements have occurred in incandescent gas lighting as well. The immediate effect has been to alter the relative amount of heat production which accompanies the light, and, therefore, entirely to change their relative ventilating value. Generally speaking, the new incandescent lighting is hotter and the combustion of the gas constituents far more perfect. On the other hand, the heat production of the new globes is very much lower. Therefore to-day the difference in their heat ventilating powers is greatly increased, so that some definite comparative experiments with the *older* types serve a particularly useful purpose, in that the preference they give to gas lighting is in reality increased with the "improved" lighting units which to-day are in operation.

### A Comparison.

"Two 25-candle-power electric lamps at that time (1908) gave an hourly production of 658 thermal units, whilst gas to produce the same amount of light evolved 1,382 thermal units, and, in addition, some 1,012 grains of water vapour and 1.54 cubic feet of carbonic acid gas. These products are given off above the inmates of the room and therefore do not vitiate the atmosphere, as is commonly supposed. The greater heat from the gas pulls the lower air to the ceiling, and the heat and moisture are absorbed by the walls and the ceiling. If there are, say, six persons in the room lit in this way, the heat given out by *them* is nearly twice that of the gas burners, *viz.*, 2,040 thermal units, and the water vapour from the skin and breath more than four times that formed by the gas burning, *viz.*, 4,200 grams. Similarly, the carbonic acid evolved by breathing amounts to 4.8 cubic feet, as against the 1.54 cubic feet from the gas lamps. There are thus in the lower parts of the room products of combustion to be removed far in excess of those produced by the lighting, and thus it follows that the increased heating effect of the gas lighting is an aid to the ventilation of the room, a matter of some importance in small rooms, and places gas at a distinct advantage over the colder electric lighting."

### The Deduction.

The natural comment upon this statement will be that the gas-burner is consuming the oxygen of the air, and it is true that if the combustion took place in a sealed chamber, the oxygen would be quickly exhausted and the products accumulate to an indefinite extent. But in practice there are innumerable inlets and exits for air, more in some tenement houses than comfort alone permits, and the conditions of the closed chamber are not even distantly approached in any actual room. In practice from a great number of experiments, Dr. Rideal affirms that with room conditions and ventilators of the minimum standards which would be accepted in modern times, the proportion of carbonic acid in the air is practically the same for both illuminants. In other words, the ventilating effect of the gas-burners is amply sufficient to overcome any specific vitiating effect they may themselves produce. "It is, therefore, not surprising that the search for the physiological effects of lighting by gas has been in the main a fruitless one."

### Actual Illumination.

By a further set of observations, 6,000 in all, it was sought to discover whether any falling-off in condition or working capacity could be detected when the two different illuminants were used in equal intensity, and it is perhaps not surprising that, with very few exceptions, these failed absolutely to show any measurable advantage for either. Also, providing average and not elaborate ventilation be provided, and the customary public health regulations as to space per individual are observed, the capacity of the persons exposed showed no deviation from the normal. Between the two lights "the differences found were so small as to be absolutely devoid of any hygienic significance; they were, in fact, much less than are constantly produced in healthy subjects by slight changes in their condition or environment."

### Conclusions.

Granting, therefore, the better ventilation provided by gas, also realising the now-accepted fact

**Health and Artificial Lighting**—(*continued*).

that carbon-dioxide, as such in the air, has not nearly the highly injurious effect formerly attributed to it, and remembering that the products heat, carbonic acid, and moisture, so far as they modify the health of people within the room, are derived from the inmates themselves, rather than the illuminant, then it is obvious that it is more than possible that we are at times inclined to lay far too much stress upon the necessity for doing away with gas-burners and installing the accepted electric system. On the whole, medical conclusions are completely in accord with Dr. Rideal's findings, when the broad issue is considered and not the special case. A point is also made in the suggestion that "the economic advantages to be derived from ventilation by gas, in the new housing schemes, by reducing the amount of material in chimneys, with the old fashioned domestic coal fire, should be regarded as of national importance at the present time." Indeed, the coal fire itself, as England knows it, has many critics.

